# The effect of replacing grains with quinoa on cardiometabolic risk factors and liver function in patients with non-alcoholic fatty liver: a randomized-controlled clinical trial

**DOI:** 10.3389/fnut.2025.1505183

**Published:** 2025-03-03

**Authors:** Afsane Gholamrezayi, Somayeh Hosseinpour-Niazi, Parvin Mirmiran, Azita Hekmatdoost

**Affiliations:** ^1^Department of Clinical Nutrition and Dietetics, Faculty of Nutrition Sciences and Food Technology, National Nutrition and Food Technology Research Institute, Shahid Beheshti University of Medical Sciences, Tehran, Iran; ^2^Nutrition and Endocrine Research Center, Research Institute for Endocrine Sciences, Shahid Beheshti University of Medical Sciences, Tehran, Iran

**Keywords:** quinoa, NAFLD, liver function, lipid profile, randomized controlled trial, cardiovascular disease

## Abstract

**Purpose:**

Quinoa is a food containing dietary fiber and various phytochemicals with high nutritional value, which has a structure similar to whole grains. This randomized controlled trial aimed to assess the effect of substituting grains with quinoa on cardiovascular risk factors and liver function in individuals with Non-alcoholic fatty liver disease (NAFLD).

**Methods:**

Forty-six participants were randomly assigned to either a control group, which maintained their regular grain-based diet, or an intervention group, where grains were replaced with quinoa for 12 weeks. Participants in the quinoa group were instructed to substitute grains with quinoa during lunch for 12 weeks. The primary outcome was to assess the changes in the Controlled Attenuation Parameter (CAP) score between the intervention and control groups. Secondary outcomes included the difference in cardiometabolic risk factors and liver function between the two groups.

**Results:**

Following 12 weeks of intervention with quinoa, a significant reduction in weight, and waist circumferences (WC) were observed compared to the control group (*p* value < 0.05). Furthermore, even after adjustment for weight change, there was a significant reduction in CAP score, serum levels of low-density lipoprotein cholesterol (LDL-C), and an improvement in homeostatic model assessment for insulin resistance (HOMA-IR) in the quinoa group compared to the control group after the 12 weeks (*p* value < 0.05). However, no significant changes were observed in other measured parameters, including liver enzymes, fibroscan, fasting plasma glucose, total cholesterol (TC), high-density lipoprotein cholesterol (HDL-C), and inflammatory factors.

**Conclusion:**

This study demonstrated that replacing grains with quinoa led to a significant improvement in the CAP score, HOMA-IR, and LDL-C in individuals with NAFLD, regardless of any weight changes. Thus, incorporating quinoa—a plentiful and low-cost source of bioactive compounds—into the diets of NAFLS patients as a staple food could improve several cardiometabolic risk factors in these individuals.

**Clinical Trial Registration:**

IRCT20100524004010N37.

## Introduction

Non-alcoholic fatty liver disease (NAFLD) is one of the most prevalent liver diseases in the world. NAFLD includes a wide range of pathological conditions, from simple hepatic steatosis to nonalcoholic steatohepatitis (NASH). Simple hepatic steatosis is characterized by a high accumulation of triglycerides (TG) in more than 5% of liver weight/volume, while NASH involves liver cell inflammation and destruction that can progress to cirrhosis and hepatocellular carcinoma (1). The worldwide prevalence of NAFLD is estimated at 25%, with this number steadily rising due to the obesity epidemic (2). NAFLD often co-occurs with metabolic syndrome manifestations in the liver, such as dyslipidemia, insulin resistance, obesity, and hypertension (3). Pathological factors like insulin resistance, lipid metabolism dysfunction, oxidative stress, inflammation, apoptosis, and fibrosis are closely associated with NAFLD (4). This condition is recognized as a leading cause of mortality from liver diseases (5).

The main risk factors associated with this condition involve a diet rich in fat, excessive consumption of simple sugar, and consuming large meals close to bedtime (2). Treatment strategies for managing NAFLD include a combination of pharmacological and non-pharmacological approaches. Lifestyle modifications, maintaining healthy dietary habits, weight reduction for overweight individuals, and consistent physical activity are among the most successful interventions for NAFLD (6, 7). Studies have indicated that a diet high in antioxidants can be an effective treatment for NAFLD (8).

Quinoa, scientifically known as *Chenopodium quinoa*, has gained significant popularity in European, African, and North American countries in recent times (9). It is recognized as a valuable source of phytochemicals with antioxidant properties, including flavonoids, phenolic acids, and fat-soluble vitamins (10). Quinoa boasts a higher quantity and quality of protein compared to other grains and is gluten-free, easily digestible, and rich in protein content (11). Additionally, it has a low glycemic index, an optimal omega-6 to omega-3 ratio, 10% dietary fiber, and is abundant in vitamins such as riboflavin, folic acid, and thiamine, surpassing rice in these nutrients (11, 12). Its nutritional and biological characteristics have led to its designation as “one of the grains of the 21st century,” with documented beneficial effects on obesity, cancer, diabetes, immune regulation, and cholesterol reduction (13). Research suggests that the favorable properties of quinoa may influence various metabolic factors, potentially benefiting individuals with conditions like obesity and type 2 diabetes (14). Additionally, another study involving quinoa in a high-fat diet in rats showed improvement in hepatic steatosis, oxidative stress, and inflammatory responses, along with reduced levels of non-esterified fatty acids in the liver and adipose tissue (15). Therefore, it seems that all these beneficial factors in quinoa may have positive health effects on many metabolic factors. Some evidence and human studies on obese individuals and those with type 2 diabetes indicate the potentially beneficial effects of quinoa on metabolic factors involved in the pathogenesis of NAFLD disease (16–18). Animal studies have indicated that quinoa consumption can lower total cholesterol (TC), low-density lipoprotein cholesterol (LDL-C), liver TG, liver enzymes aspartate transaminase (AST) and alanine transaminase (ALT), and malondialdehyde levels, as well as mitigate liver damage (19, 20).

While human studies investigating the effects of quinoa on NAFLD patients are lacking, existing research on other populations has yielded conflicting results. This study aims to investigate the effects of quinoa consumption on cardiovascular risk factors and liver function in individuals with NAFLD.

## Materials and methods

### Participants and study design

This is a randomized controlled trial (RCT). This RCT was registered in the Iranian Registry of Clinical Trials (IRCT) (code: IRCT20100524004010N37).^1^ The Ethics Committee of Shahid Beheshti University of Medical Sciences approved the study. At the commencement of the trial, written informed consent was obtained from all subjects.

Of the participants who attended the clinic Gastroenterology and Hepatology at hospitals affiliated with Shahid Beheshti University of Medical Sciences, Tehran, from July 23, 2023, to October 25, 2023. A total of 115 NAFLD subjects were screened. Diagnosis of NAFLD was performed according to the criteria of the American Gastroenterology Association (21), including evidence of liver steatosis based on liver elastography (grade 1 to 3 fatty liver) and a Controlled Attenuation Parameter (CAP) score of more than 263. Eligible participants had a clinical diagnosis of NAFLD, were aged 18–50 years, and had a body mass index (BMI) of more than 25 kg/m^2^. Exclusion criteria included dietary changes due to a specific disease, weight loss of more than 5% in the last 6 months, kidney and/or liver disease (such as Wilson disease, autoimmune liver disease, hemochromatosis, viral infections, or alcoholic fatty liver), cardiovascular disease, diabetes, malignancy, thyroid disorder, autoimmune disease, and the use of hepatotoxic drugs (such as methotrexate, amiodarone, tamoxifen, nifedipine, corticosteroids, valproate, and antiviral drugs), history of smoking, drug abuse, using dietary supplements, and history of quinoa allergy.

### Randomization and allocation concealment

**Figure 1:**
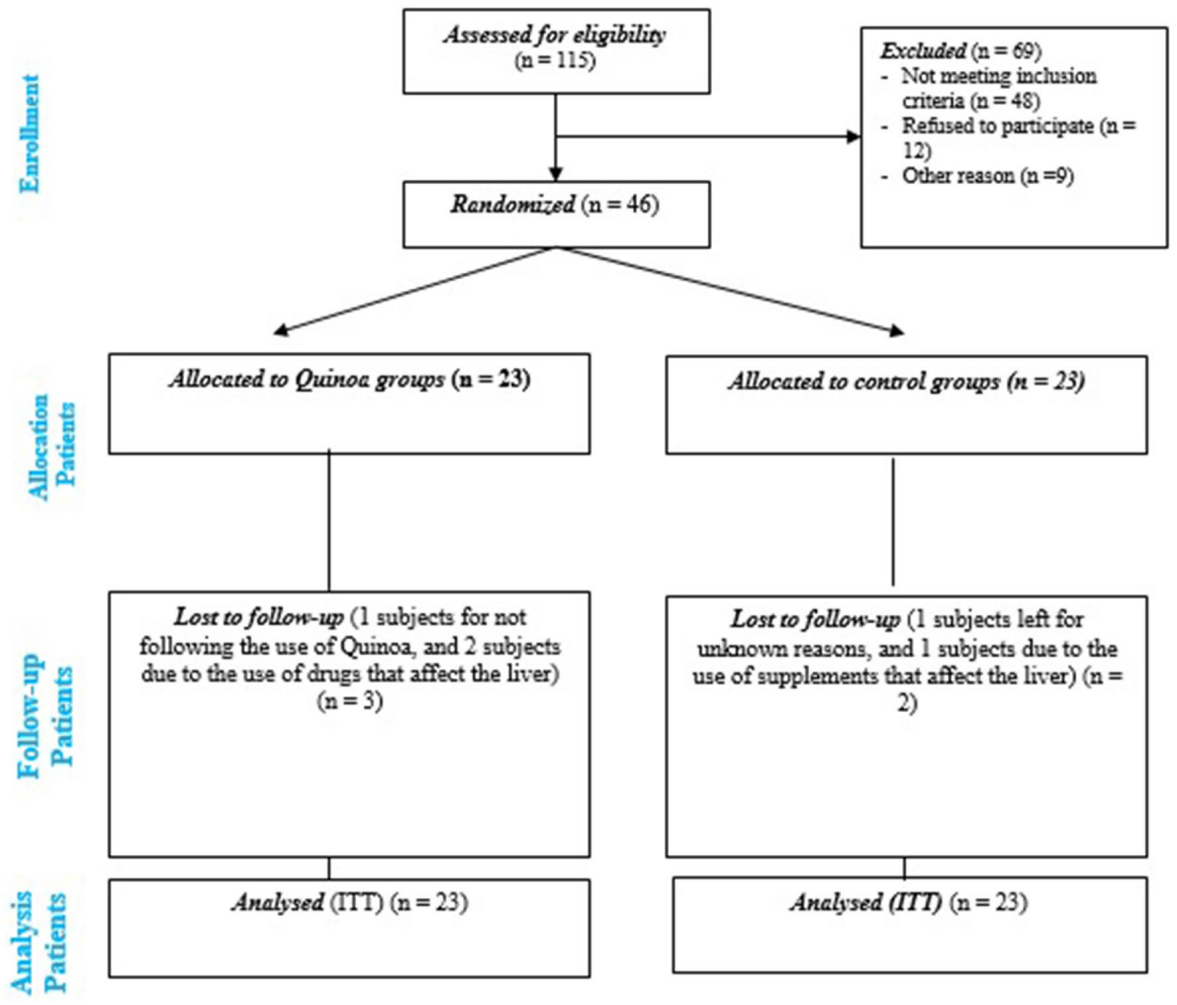
Consort flow diagram for the trial.

Permuted block randomization sequences (six participants per block) were created by the randomization website.^2^ Participants were assigned randomly (1:1 ratio) to either the quinoa group or the control group. The recruitment of participants is shown in Figure 1.

An independent staff member randomly assigned the participants to one of the two interventions. The treatment allocation was concealed from all researchers using sequentially numbered sealed opaque envelopes. These envelopes were opened sequentially in the presence of participants during their initial visit.

### Blinding

In the current study, regarding the type of interventions, blinding of participants to their group allocation was not achievable. Nevertheless, before enrollment, participants were unaware of their group assignments. The researcher and laboratory technicians evaluating the outcome were kept blind to the intervention sequences.

### Dietary interventions

At the beginning of the study, the objectives were explained to the participants and general recommendations regarding healthy food intake were provided for 2 weeks (run-in period). Eligible participants were randomly allocated to the quinoa group or control group over 12 weeks. Participants in the quinoa group were instructed to substitute grains with quinoa during lunch for 12 weeks. Due to the participants being overweight and obese, the dietary interventions were structured to provide 500 kcal/d less than their energy requirement. The amount of macronutrients was calculated as 55% from carbohydrates, 15% from protein, and 30% from fat. The amount of quinoa consumed at lunch by each person was determined according to the calories and carbohydrates calculated based on weight, height, and gender, averaging 49.56 ± 8.77 grams in the studied population (intervention group).

The quinoa used in this study was purchased from Kara Quinoa Company, (Hamedan, Iran). The macronutrient, micronutrient, and vitamin contents of cooked quinoa are shown, respectively, in Table 1 and Supplementary Table S1. The researcher provided instructions on how to cook quinoa in the intervention group. Furthermore, participants in the control group were instructed to avoid consuming products containing quinoa throughout the study.

**Table 1:** Macro-nutrient contents of quinoa and selected foods, per 100 grams cooked weight.

	Quinoa	Bean	Maize	Rice	Wheat
Energy (Kcal/100 g)	399	367	408	372	392
Protein (g/100 g)	16.5	28.0	10.2	7.6	14.3
Fat (g/100 g)	6.3	1.1	4.7	2.2	2.3
Total carbohydrate (g/100 g)	69.0	61.2	81.1	80.4	78.4

The researcher contacted the participants weekly to monitor the consumption of quinoa and grains in the intervention and control groups, respectively. To assess the adherence to interventions, the researcher compared the intake of quinoa and grains by the participants with the dietary instructions and reinforced their dietary adherence. Non-adherence was defined as consuming less than 80% of the recommended amount of Quinoa. Additionally, dietary information was gathered using 24-h dietary recall throughout the study. The intake of macro- and micronutrients was determined using NUTRITIONIST IV version 7.0 (N-Squared Computing, Salem, OR, United States), designed for Iranian foods. Participants were instructed to maintain their level of physical activity and not alter their medications during the 12-week interventions unless advised by their healthcare providers.

### Primary and secondary outcomes

The primary outcome was the difference in the change of CAP score between the two groups from baseline until the 12-week follow-up. The secondary outcomes included changes in ALT, AST, Gamma-glutamyltransferase (GGT), Fibroscan, weight, WC, fasting blood sugar (FBS), insulin, homeostatic model assessment for insulin resistance (HOMA-IR), quantitative insulin sensitivity check index (QUICKI), High-sensitivity C-reactive protein (hs-CRP), and lipid profiles.

### Measurements

#### Demographic and dietary intake assessment

The International Physical Activity Questionnaire (IPAQ) (22) was utilized to regulate and evaluate the participants’ degree of physical activity, serving as a confounding factor in assessing physical activity levels. The participants’ physical activity, as measured by this questionnaire, was assessed at the beginning and end of the study.

#### Anthropometric assessment

Weight was assessed using a Seca portable digital scale manufactured in Germany, which has a precision of 100 g. The measurement was taken with minimum clothing and without wearing shoes. The height was determined using a stadiometer, which has a precision of 0.5 cm, and the measurement was taken without wearing shoes. BMI was computed using the formula: weight (in kilograms) divided by height squared (in meters). The waist circumference (WC) were measured using a Seca waist measuring instrument, namely in the central area between the iliac crest and the final rib.

At the baseline and the 12-week follow-up, following a fasting period of 10–12 h, the laboratory technician collected 10 mL of venous blood from the participants. Following coagulation in the surroundings, the serum was promptly separated using centrifugation and stored at a temperature of −70°C until it was dispatched to the laboratory for analysis. The liver enzymes ALT, AST, and GGT, as well as high-density lipoprotein cholesterol (HDL-C), TG, and FBS content were assessed using a Pars Azmon Company kit (Pars Azmon, Tehran, Iran) and an enzymatic colorimetric approach. The Pars test kit utilized enzyme photometry to quantify the levels of TC (Pars Azmon, Tehran, Iran). LDL-C concentration was also calculated using Friedewald formula (23): LDL-C (mg/dL) = TC (mg/dL) − HDL-C (mg/dL) − TG (mg/dL)/5. Serum insulin concentration was measured using a commercial enzyme-linked immunosorbent assay (ELISA) kit (DiaSorin ELISA kit, Italy, REF 310360).

HOMA-IR (insulin resistance index) and QUICKI (insulin sensitivity index) indices were calculated using the following formulas.

$\mathrm{HOMA}-IR=\left[FBS\;\left(\mathrm{mg}/\mathrm{dl}\right)\times \mathrm{Fasting Insulin}\;\left(\mu U/\mathrm{ml}\right)\right]/405$ (24).

$\mathrm{QUICKI}=1/\left[\log\;\mathrm{Fasting Insulin}\;\left(\mu U/\mathrm{ml}\right)+\log\;FBS\;\left(\mathrm{mg}/\mathrm{dl}\right)\right]$ (24).

The manufacturer’s instructions were followed to measure serum levels of hs-CRP using a colorimetric enzyme-linked immunosorbent assay (R&D Systems, Minneapolis, MN).

Changes in liver function and liver fibrosis were also performed using fibroscan under the supervision of a gastroenterology and liver specialist.

#### Statistical analysis methods

All analyses were conducted using Stata software version 14.0 (StataCorp LLC, TX, United States). In the current study, 42 participants were required to detect (α error = 0.05, β error = 0.20) differences in a 25 IU/L reduction in ALT between quinoa and control groups (25). Accounting for an attrition rate of 10%, finally 23 participants were included in each group.

All participants who were randomly assigned to the dietary interventions underwent analyses following the intention-to-treat (ITT) principle. The Multiple imputation, Chained Equations (MICD) procedure was used to impute missing data for both primary and secondary outcomes for the 5 participants who withdrew from the study. The predictors in the multiple imputation process encompassed all variables listed in Table 2.

**Table 2:** Baseline characteristics of participants according to group of intervention.

	Quinoa group (*n* = 23)	Control group (*n* = 23)	*p* value
Age, y	39.6 ± 5.1	39.9 ± 5.5	0.884
Male, *n* (%)	19 (51.4)	18 (48.6)	0.500
Weight, Kg	92.3 ± 12.0	92.5 ± 11.1	0.976
BMI, kg/m^2^	29.9 ± 5.1	31.7 ± 5.1	0.248
Waist circumference, Cm	111.3 ± 7.7	109.5 ± 7.8	0.421
Physical activity, Met. h/wk	30.5 ± 4.3	30.6 ± 4.7	0.874
CAP score	315 ± 35	315 ± 36	0.949
FPS, mg/dl	94.3 ± 10.2	98.8 ± 10.7	0.158
HOMA-IR	3.7 ± 1.9	3.5 ± 1.8	0.724
QUICKI	0.32 ± 0.021	0.32 ± 0.023	0.685
Insulin	15.8 ± 7.4	14.4 ± 6.9	0.508
ALT, IU/L	35.6 ± 13.1	33.1 ± 11.4	0.495
AST, IU/L	30.6 ± 8.8	30.6 ± 9.7	0.892
GGT, IU/L	33.3 ± 17.7	34.2 ± 14.2	0.872
TC, mg/dl	186 ± 29	190 ± 29	0.624
TG, mg/dl	177 ± 48	176 ± 57	0.964
HDL-C, mg/dl	39.1 ± 3.7	39.4 ± 6.5	0.824
LDL-C, mg/dl	116 ± 26.2	119 ± 25.6	0.696
FibroScan	5.9 ± 2.1	6.2 ± 2.1	0.663
hs-CRP, mg/L	4.2 ± 3.3	4.1 ± 2.9	0.942

Data are mean ± SD unless otherwise indicated.

FPS, fasting plasma glucose; BMI: Body mass index; WC: waist circumference; CAP; Controlled Attenuation Parameter; HOMA-IR, Homeostatic Model Assessment for Insulin Resistance; QUICKI, Quantitative insulin sensitivity check index; ALT, Alanine transaminase; AST, aspartate aminotransferase; GGT, gamma-glutamyl transferase; TC, total cholesterol; TG, triglycerides, HDL-C, high-density lipoprotein cholesterol; LDL-C, low-density lipoprotein cholesterol; hs-CRP, High sensitivity C-reactive protein.

The demographic variables and dietary variables are reported as mean ± standard deviation (SD), and dichotomous variables as count (percentage) in the baseline characteristics. The histograms and the Shapiro–Wilk test were used to evaluate the normal distribution of primary and secondary outcomes. Analysis of covariance (ANCOVA), with adjustment for baseline values (model 1) and weight change (model 2), was employed to compare the effects of quinoa versus the control group on changes in the primary and secondary outcomes. All statistical tests were considered statistically significant when the *p* value was <0.05.

## Results

### Characteristics of the participants

This RCT was conducted from July 23, 2023, to October 25, 2023. A total of 46 eligible participants with NAFLD were randomly assigned to either quinoa (*n* = 23) or control (*n* = 23) groups. Five participants withdrew from the study. Finally, all patients (23 in the quinoa and 23 in the control groups) entered the analysis with intention-to-treat (ITT) analysis (Figure 1).

Table 2 presents the baseline characteristics of participants. There were no significant differences observed between the quinoa and control groups in terms of basic characteristics including sex, age, physical activity level, anthropometric characteristics, liver enzymes, liver function, glycaemic status, and lipid profile. The mean age and BMI of the participants were 39.6 ± 5.1 years and 32.2 ± 4.4 kg/m^2^ in the quinoa group and 39.9 ± 8.5 years and 31.7 ± 5.1 kg/m^2^ in the control group, respectively.

The dietary intake of macronutrients and micronutrients for participants in both the quinoa and control groups is presented in Table 3. At the end of the follow-up period, both the Quina and control groups showed a decrease in energy and carbohydrate intake, along with an increase in Vitamin E intake. In the quinoa group, fat and omega-6 consumption decreased, while omega-3 intake increased at the end of intervention. There were no significant differences found in the intake of protein, saturated fatty acid (SFA), cholesterol, fiber, magnesium, and vitamins at the end of the study in both groups.

**Table 3:** Dietary intake of the participants according to quinoa and control groups.

	Quinoa			Control			*p* value^b^
	Baseline	After 12	*p* value^a^	Baseline	After	*p* value^a^	
Energy (Kcal/d)	**2,359** ± **473**	**2089.3** ± **360.4**	**<0.001**	**2,526** ± **740.8**	**2,240** ± **791**	**0.003**	0.420
Carbohydrate (g/d)	**298** ± **77.5**	**259.12** ± **55.4**	**0.001**	**338** ± **127**	**265** ± **112**	**<0.001**	0.822
Protein (g/d)	94.2 ± 21.6	92.8 ± 21.8	0.739	97.2 ± 35.1	95.1 ± 25.8	0.304	0.762
Fat (g/d)	**92.7** ± **17.3**	**79.8** ± **15.9**	**0.002**	95.4 ± 30.9	84.8 ± 50.5	0.116	0.662
SFA (g/d)	23.6 ± 5.0	21.8 ± 5.0	0.098	30.3 ± 27.3	27.5 ± 21.0	0.353	0.221
MUFA (g/d)	41.8 ± 40.2	30.1 ± 7.7	0.188	29.4 ± 6.9	26.9 ± 19.3	0.640	0.475
Cholesterol (mg/d)	254 ± 85.7	246 ± 83.6	0.731	263 ± 163.5	228 ± 142.4	0.163	0.602
Fiber (g/d)	27.1 ± 7.3	25.5 ± 6.5	0.325	26.3 ± 11.9	26.8 ± 14.1	0.601	0.692
Omega 3 (mg/d)	**1.2** ± **0.6**	**3.5** ± **11.2**	**<0.001**	1.41 ± 1.0	1.26 ± 1.2	0.676	0.401
Omega 6 (mg/d)	**7.8** ± **2.4**	**6.2** ± **2.3**	**<0.001**	15.9 ± 28.1	14.2 ± 23.1	0.125	0.110
Magnesium (mg/d)	278 ± 139	270 ± 137	0.317	245.7 ± 112.6	239.4 ± 102.6	0.700	0.229
Vitamin A (RE)	945 ± 157	1,022 ± 162	0.312	894 ± 490	963 ± 318	0.447	0.593
Vitamin E (mg/d)	**8.3** ± **6.1**	**10.4** ± **4.9**	**0.036**	**9.8** ± **7.4**	**11.4** ± **5**	**0.042**	0.810
Vitamin C (mg/d)	90.8 ± 43	90.1 ± 54	0.701	86.1 ± 35.3	97.6 ± 33.3	0.670	0.502
Vitamin D (mcg/d)	8.7 ± 6	9.1 ± 3.6	0.481	8.8 ± 5.3	9.6 ± 5.7	0.268	0.471

Data are expressed as Mean ± SD.

PUFA, Polyunsaturated fatty acid; SFA, Saturated fatty acid; MUFA, Monounsaturated fatty acid.

a *p-values for comparison of within-group differences.*

b *p-values for comparison of mean values between two groups.*

Bold values are significant.

#### Primary outcomes

A reduction in CAP score was observed at week 12 in the quinoa group after adjustment for baseline value. The mean difference ± SD in change was −32.3 ± 6.2 in the quinoa group compared to −13.8 ± 6.2 in the control group, with a *p* value of 0.044. The difference in change of CAP score between the groups remained significant, after adjusting for weight change (−29.0 ± 6.4 in the quinoa group vs. 12.2 ± 6.3 in the control group; *p* value = 0.039) (Table 4).

**Table 4:** The 12-week change in anthropometric characteristics, liver enzymes, liver function, glycaemic indices, and lipid profile after the quinoa and control groups.

	Quinoa group (*n* = 23)	Control group (*n* = 23)	*p* value
**Primary outcome**			
**CAP score**			
Model 1	**−32.3 ± 6.2**	**−13.8 ± 6.2**	**0.044**
Model 2	**−29.0 ± 6.4**	**−12.2 ± 6.3**	**0.039**
**Secondary outcomes**			
**Weight, Kg**			
Model 1	**−3.1 ± 0.7**	−0.5 **± 0.7**	**0.017**
**WC, Cm**			
Model 1	**−2.3 ± 0.6**	−0.5 **±** 0.6	**0.035**
**Liver enzyme and liver function**			
**ALT, IU/L**			
Model 1	**−8.22** ± 2.1	−2.69 ± 2.1	0.081
Model 2	**−7.32** ± 2.2	−3.59 ± 2.2	0.251
**AST, IU/L**			
Model 1	−6.96 ± 3.0	−0.16 ± 3.0	0.121
Model 2	−7.42 ± 3.1	0.30 ± 3.1	0.101
**FibroScan**			
Model 1	**−0.60 ± 0.2**	−0.12 **± 0.2**	0.061
Model 2	−0.56 ± 0.2	−0.17 ± 0.2	0.146
**GGT, IU/L**			
Model 1	−0.28 ± 2.8	1.52 ± 2.8	0.655
Model 2	0.72 ± 2.8	0.51 ± 2.8	0.961
**Glycaemic indices**			
**FPG, mg/dL**			
Model 1	−1.6 ± 2.0	1.9 ± 2.1	0.230
Model 2	−1.7 ± 2.1	2.1 ± 2.1	0.226
**HOMA-IR**			
Model 1	**−0.97 ± 0.23**	**−0.03 ± 0.23**	**0.009**
Model 2	**−0.85 ± 0.24**	**−0.15 ± 0.24**	**0.050**
**QUICKI**			
Model 1	0.011 ± 0.01	0.006 ± 0.01	0.530
Model 2	0.012 ± 0.01	0.005 ± 0.01	0.409
**Insulin**			
Model 1	**−3.65 ± 0.9**	−0.50 **± 0.9**	**0.021**
Model 2	**−3.14 ± 0.9**	−1.00 **± 0.9**	0.106
**Lipid profile**			
**TC, mg/dL**			
Model 1	−8.46 ± 4.2	−1.06 ± 4.2	0.222
Model 2	−7.83 ± 4.3	−1.69 ± 4.3	0.343
**LDL-C, mg/dL**			
Model 1	**−13.83 ± 3.7**	**2.19 ± 3.7**	**0.005**
Model 2	**−12.81 ± 3.9**	**1.18 ± 3.9**	**0.018**
**TG, mg/dL**			
Model 1	**−17.2 ± 6.1**	3.1 **± 6.1**	**0.024**
Model 2	**−15.9 ± 6.3**	1.6 **± 6.3**	0.063
**HDL**-**C, mg/dL**			
Model 1	−0.39 ± 0.4	−0.19 ± 0.4	0.753
Model 2	−0.25 ± 0.4	−0.33 ± 0.4	0.898
**Inflammatory marker**			
**hs-CRP, mg/L**			
Model 1	0.32 ± 0.4	0.62 ± 0.4	0.565
Model 2	0.32 ± 0.4	0.62 ± 0.4	0.594

Data are expressed as Mean ± SEM.

Significant data is bolded.

FPS, fasting plasma glucose; BMI, Body mass index; WC, waist circumference; CAP, Controlled Attenuation Parameter; HOMA-IR, Homeostatic Model Assessment for Insulin Resistance; QUICKI, Quantitative insulin sensitivity check index; ALT, Alanine transaminase; AST, aspartate aminotransferase; GGT, gamma-glutamyl transferase; TC, total cholesterol; TG, triglycerides, HDL-C, high-density lipoprotein cholesterol; LDL-C, low-density lipoprotein cholesterol; hs-CRP, High sensitivity C-reactive protein.

Model 1 adjusted for baseline values.

Model 2 adjusted for baseline values and weight change.

#### Secondary outcomes

Based on the results presented in Table 4, at week 12, the quinoa group exhibited decreases in ALT (−7.32 ± 2.2 in the quinoa group vs. −3.59 ± 2.2 in the control group; *p* value = 0.251), AST (−7.42 ± 3.1 in the quinoa group vs. 0.30 ± 3.1 in the control group; *p* value = 0.101), and fibroScan (−0.56 ± 0.2 in the quinoa group vs. −0.17 ± 0.2 in the control group; *p* value = 0.146); however, the difference between the two groups was not significant.

Additionally, within the quinoa group, significant decreases were observed in HOMA-IR (−0.97 ± 0.23 in the quinoa group vs. -0.03 ± 0.23 in the control group; *p* value = 0.009) and insulin concentration (−3.65 ± 0.9 in the quinoa group vs. −0.50 ± 0.9 in the control group; *p* value = 0.021). However, after adjustment for baseline value and weight change, only HOMA-IR displayed a reduction after 12 weeks of quinoa intervention, when compared to the control group (−0.85 ± 0.24 in the quinoa group vs. −0.15 ± 0.24 in the control group; *p* value = 0.050).

Furthermore, significant reductions in TG (−17.2 ± 6.1 in the quinoa group vs. 3.1 ± 6.1 in the control group; *p* value = 0.024) and LDL-C (−13.83 ± 0.3.7 in the quinoa group vs. 2.19 ± 3.7 in the control group; *p* value = 0.005) levels were noted in the quinoa group, compared to the control group. However, after adjustment for baseline value and weight change, only LDL-C displayed a reduction after 12 weeks of quinoa intervention, when compared to the control group (−12.81 ± 3.9 in the quinoa group vs. 1.18 ± 3.9 in the control group; *p* value = 0.018).

Both weight (−3.1 ± 0.7 in the quinoa group vs. -0.5 ± 0.7 in the control group; *p* value = 0.017) and WC (−2.3 ± 0.6 in the quinoa group vs. −0.5 ± 0.6 in the control group; *p* value = 0.035) decreased following quinoa consumption, and the difference between the two dietary interventions was significant. Lastly, no significant difference in hs-CRP concentration was reported at week 12 in either quinoa or control groups (Table 4).

## Discussion

To our knowledge, this study is the first randomized control trial that has assessed the effects of substituting lunch grains with quinoa on obesity indicators, lipid profile, glycemic status, and liver function in patients with NAFLD. This study revealed that the substitution of grains with quinoa significantly improved the CAP score, HOMA-IR, and LDL-C in NAFLD subjects, independent of weight change.

The findings of our study showed a significant decrease in CAP score after 12 -weeks of intervention with quinoa compared to the control group. However, we did not observe beneficial or significant effects on liver enzymes and fibroscan. Despite the potential benefits of quinoa on liver tissue function, it does not seem to reduce inflammatory processes caused by elevated liver enzyme levels. Our findings are clinically significant as a CAP score above 280 or 290 dB/m indicates severe steatosis with a 22% prevalence of increased liver stiffness in subjects with metabolic risk factors, while a CAP score between 248 and 290 dB/m is associated with only a 5% prevalence of increased liver stiffness (26). To our knowledge, no human studies have been conducted to investigate these aspects of liver function, with current studies limited to animal studies. For instance, a study by Song et al. in 2021 investigated the effect of feeding varying amounts of quinoa (300 grams per day) for 12 weeks in male rats with fatty liver (19). The results showed reduced TG and TC levels in the liver, decreased liver damage, increased antioxidant activities, and overall prevention of NAFLD by controlling body weight, reducing oxidative stress, and regulating lipid metabolism and immune response gene expression (19). The relatively low levels of liver enzymes may explain the modest effect of this intervention. Additionally, the small average intake of quinoa (about 49 grams) compared to the animal study may also contribute to these findings.

Furthermore, significant and decreasing changes were shown in all anthropometric factors, including weight, and WC, in the quinoa group compared to the control group. As weight loss interventions are considered crucial in the treatment of certain conditions, these findings may contribute to improving various pathogenic processes associated with the disease (21). Evidence suggests that a weight loss of at least 5% of body weight is necessary to enhance histological and functional liver symptoms. The observed weight loss of approximately 3% following quinoa intervention could explain some of our results, such as the lack of significant effects on liver enzymes (21). Therefore, combining quinoa intervention with weight loss regimes may enhance treatment outcomes and improve patients’ adherence to weight loss protocols. A meta-analysis conducted in 2021 on five RCT studies with a total of 206 participants, revealed that supplementation with quinoa seeds led to a significant reduction in weight, WC, and fat mass (27). However, no significant effect on BMI reduction was reported, possibly due to the limited number of studies and also some trials involving individuals with normal weight. Laboratory studies suggest that phytoectosteroids, particularly 20-hydroxyecdysone, play a key role in the weight loss mechanism induced by quinoa consumption. These compounds are believed to reduce the size and storage capacity of fat cells, downregulate genes involved in fat accumulation such as lipoprotein lipase, and modulate related to inflammatory adipokines (28, 29). Several mechanisms are proposed to be involved in this weight loss process, including favorable alterations in hormone level that influence appetite regulation, such as leptin and ghrelin (30). Additionally, quinoa’s high content of soluble and insoluble fiber may increase satiety and correct intestinal dysbiosis (31, 32). Furthermore, the presence of quinoa saponins is thought to reduce systematic inflammation (33). These combined mechanisms highlight the potential of quinoa as a beneficial dietary component for weight management and overall health.

The current study’s findings indicate that, except for HOMA-IR, there were no significant differences in glycemic indices after 12 weeks of substituting lunch grains with quinoa compared to the control group. Similar results were also reported in other studies. For instance, a prospective and double-blind study involving 35 overweight women found no significant effect on FBS when comparing the group consuming 25 grams of quinoa flakes to those having corn flakes after 4 weeks of intervention (34). Another study in Brazil involving students aged 18–45 years did not show significant effects on glycemic index after a 30-day intervention with quinoa (35). Furthermore, a RCT with a parallel design investigating the effects of 25 and 50 grams of quinoa per day on 50 overweight and obese participants over 12 weeks did not report significant effects on FBS and insulin levels (18). Overall, evidence suggests that the major effect of quinoa on glycemic status is related to postprandial glucose response and enhanced insulin sensitivity (17). Compounds like 20-hydroxyecdysone and polyphenols, particularly flavonoids present in quinoa, may increase insulin sensitivity and improve hepatic gluconeogenesis by affecting PI3K-dependent insulin signaling pathways (29, 36). In addition, the high fiber content and low glycemic index of quinoa compared to other grains may also contribute to these beneficial effects (34).

After 12 weeks of intervention with quinoa compared to the control group, there was a significant decrease in serum TG and LDL-C levels. The effect of quinoa on TG concentration disappeared after adjustment for weight change. Consistent with our findings, various studies have reported similar results showing a significant reduction in TG and no significant impact on serum HDL-C following quinoa intervention (16, 18, 27, 34). However, conflicting results have been reported regarding TC and LDL-C levels. For instance, a comprehensive study demonstrated a decrease in both factors after quinoa consumption (27), while a study involving obese and overweight individuals did not show significant effects after a 12-week intervention (18). These contradictory results can be caused by the variety in the type of quinoa-containing products, the dosage administered to participants, and notably, the variation in baseline levels of these factors across studies. The beneficial effects of quinoa on lipid profile levels may be attributed to its high fiber content, and the presence of compounds such as 20-hydroxyecdysone, polyphenols, and phytosterols, which are key factors in reducing blood lipid levels (27). Additionally, the protein isolated from quinoa could play a role in lowering cholesterol by reducing the expression of hepatic 3-hydroxy-3-methylglutaryl coenzyme A (HMG-CoA) reductase and increasing bile acid excretion from the intestine (37).

According to comprehensive review (38), there is no evidence that quinoa has a different effect on cardiovascular risk factors between men and women. Therefore, gender is not a relevant variable in this study and we did not perform analyses based on gender.

One of the most important strengths of this study was the interpretation of the findings based on the ITT principles, a low dropout rate, and a RCT design that allowed for controlling the confounders. Additionally, this study was the first human investigation into the potential benefits of substituting lunch grains with quinoa for patients with NAFLD. However, several limitations warrant consideration. The assessment of adherence to dietary interventions relied on self-report diet records, and due to limited funding, we were unable to measure the effective amount of bioactive substances in quinoa to assess adherence accurately. To address this, a dietitian contacted participants weekly to reinforce adherence to dietary recommendations. Another limitation was the lack of blinding participants to the study objectives, potentially influencing their behaviors. Furthermore, not conducting liver biopsies, the gold standard for NAFLD treatment assessment, was another constraint in the current study.

## Conclusion

The findings of our study indicate that substituting quinoa for traditional lunch grains may have a beneficial effect on weight management, insulin resistance, and LDL-C levels. Thus, incorporating quinoa—a plentiful and low-cost source of bioactive compounds—into the diets of NAFLS patients as a staple food could improve several cardiometabolic risk factors in these individuals. However, additional high-quality studies with larger sample sizes, as well as investigation into the bioactive components of quinoa, are necessary to validate and strengthen our results.

## Data Availability

The raw data supporting the conclusions of this article will be made available by the authors, without undue reservation.

## Footnotes

^1^ https://en.irct.ir/trial/37196

^2^ http://www.randomization.com
