# Supplementary material for: The effect of replacing grains with quinoa on cardiometabolic risk factors and liver function in patients with non-alcoholic fatty liver: a randomized-controlled clinical trial
Source: Front Nutr. 2025 Mar 3;12:1505183. doi: 10.3389/fnut.2025.1505183 (PMC11911194; doi:10.3389/fnut.2025.1505183)
Supplement: Supplementary file 1 [file Table_1.docx]

| **Supplementary Table 1.** Micro-nutrient and vitamins contents of quinoa and rice, per 100 grams cooked weight | | |
| --- | --- | --- |
|  | Quinoa | Rice |
| Calcium (mg/100 g) | 17 | 10 |
| Iron (mg/100 g) | 1.49 | 1.2 |
| Magnesium (mg/100 g) | 64 | 12 |
| Phosphorus (mg/100 g) | 152 | 43 |
| Potassium (mg/100 g) | 172 | 35 |
| Sodium (mg/100 g) | 7 | 1 |
| Zinc (mg/100 g) | 1.09 | 0.49 |
| Copper (mg/100 g) | 0.192 | 0.069 |
| Manganese (mg/100 g) | 0.631 | 0.472 |
| Selenium (µg/100 g) | 2.8 | 7.5 |
| Thiamin (mg/100 g) | 0.107 | 0.163 |
| Riboflavin (mg/100 g) | 0.11 | 0.013 |
| Niacin (mg/100 g) | 0.412 | 1.48 |
| Vitamin B-6 (mg/100 g) | 0.123 | 0.093 |
| Folate (µg/100 g) | 42 | 58 |
| Vitamin B-12 (µg/100 g) | 0 | 0 |
| Vitamin A (IU) | 5 | 0 |
| Vitamin E: alpha-tocopherol (mg/100 g) | 0.63 | 0.04 |
| Vitamin D (IU) | 0 | 0 |
| Vitamin K (µg/100 g) | 0 | 0 |
